# Hydrocarbons Emitted by Waggle-Dancing Honey Bees Increase Forager Recruitment by Stimulating Dancing

**DOI:** 10.1371/journal.pone.0105671

**Published:** 2014-08-20

**Authors:** David C. Gilley

**Affiliations:** Department of Biology, William Paterson University, Wayne, New Jersey, United States of America; Colorado State University, United States of America

## Abstract

Hydrocarbons emitted by waggle-dancing honey bees are known to reactivate experienced foragers to visit known food sources. This study investigates whether these hydrocarbons also increase waggle-dance recruitment by observing recruitment and dancing behavior when the dance compounds are introduced into the hive. If the hydrocarbons emitted by waggle-dancing bees affect the recruitment of foragers to a food source, then the number of recruits arriving at a food source should be greater after introduction of dance compounds versus a pure-solvent control. This prediction was supported by the results of experiments in which recruits were captured at a feeder following introduction of dance-compounds into a hive. This study also tested two nonexclusive behavioral mechanism(s) by which the compounds might stimulate recruitment; 1) increased recruitment could occur by means of increasing the recruitment effectiveness of each dance and/or 2) increased recruitment could occur by increasing the intensity of waggle-dancing. These hypotheses were tested by examining video records of the dancing and recruitment behavior of individually marked bees following dance-compound introduction. Comparisons of numbers of dance followers and numbers of recruits per dance and waggle run showed no significant differences between dance-compound and solvent-control introduction, thus providing no support for the first hypothesis. Comparison of the number of waggle-dance bouts and the number of waggle runs revealed significantly more dancing during morning dance-compound introduction than morning solvent-control introduction, supporting the second hypothesis. These results suggest that the waggle-dance hydrocarbons play an important role in honey bee foraging recruitment by stimulating foragers to perform waggle dances following periods of inactivity.

## Introduction

Many eusocial insects have evolved sophisticated mechanisms for communicating to nestmates information about nutrient supplies and demands. Some of the most complex communication systems have evolved among the eusocial bees (e.g. Bombini, Meliponini, Apini) where fast and efficient allocation of foraging effort allows exploitation of spatially and temporally patchy floral food sources. The most common means of coordinating foraging activities within eusocial bee colonies is chemically, through naturally selected signals (i.e., pheromones) and informational cues (e.g., food odors) to which workers respond adaptively. Chemical signals are known to activate nestmates to depart in search of food [1], [2], [3], guide recruits to the location of the food source via aerial plumes or terrestrial trails [4], [5], [6], and mark food sources to alert nestmates that the identified site has been visited [7], [8], [9], [10], [11], [12], [13], [14], [15], [16], [17], [18], [19], [20], [21], [22], [23], [24]. Chemical cues known to be used in foraging recruitment include food-source odors which stimulate foraging and convey information about the identity of profitable food sources [25], [26], and compounds left passively by foragers visiting food sources which repel both conspecifics and heterospecifics [20], [23]. Understanding how these signals and cues interact and together affect the foraging decisions of individuals to produce adaptive colony level behavior is an area of much interest (e.g. in honey bees [27], stingless bees [28], and bumblebees [24]).

Honey bees (*Apis mellifera sp.*) provide a particularly rich and economically important example of group coordination in foraging by means of chemical signals and cues. Honey bees are known to employ chemical signals and cues for foraging-related tasks both outside and inside the nest. Outside the nest, honey bee foragers use floral and site-specific odor cues to locate food sources [25], [29], [30], [31], [32], [33], [34]. Foragers also deposit scent marks which act on other foragers as short-term repellents (thought to be 2-heptanone [14], [15], [20]), or long-term attractants (thought to be hydrocarbons from the nasanov gland and/or (Z)-11-eicosen-1-ol [8], [10], [35], [20]). Within the nest, floral odors are transmitted to nestmates via trophallaxis [36], [37], [38] and have been shown to alter foragers' later food preferences in the field [39]. Reactivation of experienced foragers to exploit food sources that were profitable in the past is accomplished both by floral cues [25], [40], [41] [42] and by hydrocarbons emitted by waggle-dancing bees [43], [44]. These hydrocarbons could have an additional role in stimulating recruitment of foragers to food sources by means of the waggle dance. This possibility is of special interest because it would place these compounds in an important role in the honey bee foraging communication system and further enrich this system as an example of group coordination through complex signaling.

The waggle-dance hydrocarbons consist of at least four compounds, two alkanes (tricosane, pentacosane) and two alkenes (Z-9-tricosene, Z-9-pentacosene), which can be detected at elevated levels in air samples from the area of a hive in which dancing and recruitment occur [43]. These compounds are known constituents of the honey bee cuticle (reviewed by [45]), and are thought to be involved in nestmate recognition (reviewed by [46]). They have been sampled from air surrounding foragers at artificial flowers [47], and within the hive are three to four times more abundant on the abdomens of waggle-dancing foragers than on returning foragers that do not perform waggle dances [43]. At least some of these compounds are behaviorally active, as introduction into the hive of a synthetic blend of three of the four compounds caused bees to exit the hive [43]. Bees exiting the hive in response to introductions of the dance-compound blend visited a feeder significantly more often than when exposed to the blend's solvent, showing that foragers' response to the dance compounds is not purely aversive and suggesting that it is an adaptive response to the hydrocarbons emitted by waggle-dancing bees [44]. Stimulation of foraging observed following introduction of dance compounds is at least partly due to reactivation of experienced but currently inactive foragers; foragers which had been trained to a feeder increased their visitation to the feeder in response to introduction of the waggle-dance compounds (versus the solvent) even when the feeder had been emptied so that recruitment within the hive was not occurring [44]. Together, the results of existing studies suggest that the hydrocarbons emitted by waggle-dancing bees function as a semiochemical that stimulates foraging by alerting nestmates to favorable foraging conditions and food availability.

The present study seeks to determine whether the hydrocarbons emitted by waggle-dancing bees play a role in waggle-dance recruitment by looking for differences in dancing and recruitment behavior when the dance compounds are introduced into a hive. If the hydrocarbons emitted by waggle-dancing bees affect the recruitment of foragers to a food source, then the number of recruits arriving at a food source should be greater following dance-compound introduction than during introduction of a pure-solvent control. The study's second goal is to determine the behavioral mechanism(s) by which the dance compounds might increase recruitment. Two nonexclusive mechanisms are hypothesized: 1) recruitment increase is caused by increased intensity of dancing (i.e., via increasing motivation of the signaler), and/or 2) recruitment increase is caused by increased recruitment effectiveness of each dance (i.e., via increasing the response of the signal receiver). This study addresses both of these hypotheses by examining video records of individually marked waggle-dancing bees before and after the introduction of the dance hydrocarbons versus a solvent control.

## Materials and Methods

### Ethics Statement

This field study was conducted on privately owned land with permission from the owner, the LANXESS Corporation. No endangered or protected species were involved in this research.

Experiments were performed during July and August over the course of two summer seasons, in 2010 and 2012, in an uncultivated field in North Haledon, New Jersey (Latitude/Longitude  =  +40°56.579′, −74°11.037′). Experiments were performed sequentially on a total of four colonies (two in 2010 and two in 2012) of approximately 3000 workers of the Italian variety (*Apis mellifera ligustica*) housed in a two-frame observation hive shaded from the sun by a canopy tarp. When installed in the observation hive, each colony was provided with one frame of brood and one frame approximately half full of honey to ensure that demand for nectar remained constant throughout each replicate of the experiment. Foragers were trained to a feeder containing unscented sugar water located approximately 120 m from the hive. Training consisted of attracting to the feeder foragers at the entrance of the hive, then gradually moving the feeder to the final location [25]. Care was taken to eliminate foragers from foreign colonies by marking the initial recruits with a dot of paint and then subsequently killing any unmarked bees that visited the feeder. After the training of the bees to the feeder, we marked each bee visiting the feeder with a unique color combination to allow individual identification of the foragers experienced with the feeder-dish food source. Fifty foragers were marked for individual identification and all bees subsequently visiting the feeder were killed to ensure that any unmarked bees arriving at the feeder during experimental trials were new recruits without prior experience foraging from the feeder.

Immediately following completion of training, marking, and culling, we began experimental trials. Three experimental trials were conducted each day, one in the morning at 10∶00, one during midday at 13∶00, and one in the afternoon at 16∶00. Each trial of the experiment began when the first bee visited the filled feeder dish, which typically occurred within two minutes of filling the dish at the feeder station. The feeder dish remained empty at all times except during experimental trials to prevent recruitment of unmarked bees or discovery by scouts. To stimulate sufficient recruitment while at the same time preventing overcrowding of the feeder dish (7 cm diameter) by recruited bees, the concentration of the sugar solution was adjusted between 30% and 50%, accounting for fluctuations in the favorability of foraging conditions. Adjustment of sugar-solution concentration among the replicates of each colony was avoided so that this variable was a covariate of colony identity; averages for each colony were: 2010A = 48%, 2010B = 45%, 2012A = 35%, 2012B = 30%. Fifteen minutes following the arrival of the first bee and for forty five minutes thereafter, we recorded the identity (either the bee's unique color combination, or “unmarked”) of each bee that visited the feeder using a digital audio recorder, which enabled us to determine the time of each bee's arrival. A visit was defined as a bee landing on the dish and extending her proboscis to the feeder reservoir. All new recruits (unmarked bees) visiting the feeder for the entire hour were captured in small Ziploc bags by placing the bag over the bee and allowing it to crawl into the bag, thus minimizing disturbance of other bees at the feeder. This procedure was usually successful, but occasionally (approximately 15% of attempts) recruits did escape or were not bagged due to simultaneous arrivals, so we cannot assume that each visit by an unmarked bee was a unique individual. For this reason, subsequent analysis uses only the number of captured bees, which must have been unique individuals.

While one experimenter worked at the feeder, another remained at the observation hive, video recording the behavior of the bees in the hive and performing substance introductions. Video recording began fifteen minutes following the arrival at the feeder of the first bee. Video records were made with a high-definition video camera (Sony HDR-SR7) zoomed to the approximate area of the “dance floor”, a 25×15 cm rectangle adjacent to the hive entrance. A wedge of wood placed under the lower frame of the hive funneled incoming workers to one side of the hive so that dances were performed almost exclusively on the camera side of the hive. To prevent glare in the image, recording was done in the dark with two LED lamps placed to illuminate the dance floor area. Dark conditions were created by placement of a large (approximately 100×50×50 cm) box over the camera, flush against the pane of the observation hive, and on top of the table on which the hive rested. This box was temporarily removed, and video recording suspended, during substance introduction, for approximately 60 seconds. Substance introduction occurred thirty minutes following the arrival at the feeder of the first bee. At this time, either 300 µl of waggle-dance compound mixture or 300 µl of pure ethyl ether was introduced into the hive. The waggle-dance compound mixture contained Z-(9)-tricosene, tricosane, and pentacosane, each at an initial concentration of 0.01 g/ml, mixed at a ratio of 1∶2∶3, respectively (this ratio produces chromatograms with peak heights that approximately match those from samples of waggle dancers [43]), and then further diluted 1∶10 in ether. The substance was introduced into the hive by pipetting 300 µl onto a glass microscope slide attached to a wooden strip, which was then immediately inserted through a notch in the Plexiglas pane of the observation hive so that it rested on the floor of the hive underneath the dance floor area and adjacent to the entrance of the hive. The slide remained in the hive until the trial was complete. To avoid contamination of the solvent slide, a separate but identical slide was used for the solvent-control and waggle-dance compound introductions. To prevent observer bias, the identity of the substance introduced in each trial was not revealed to the experimenter recording bee visits to the feeder station, nor to those later transcribing the videos. To account for time-of-day effects, the identity of the substance introduced (waggle-dance compounds or solvent) was alternated so that for each colony we had approximately the same number of morning, midday, and afternoon replicates for both solvent-control and dance-compound introductions. The procedures above (with one variation for Colony 2010A, noted below) were performed sequentially using four colonies for a total of thirty solvent control trials and thirty five waggle-dance compound trials (2010A = 7 control, 8 dance compound; 2010B = 7 control, 8 dance compound; 2012A = 9 control, 8 dance compound; 2012B = 7 control, 10 dance compound).

Following the experiments, video records of behavior on the dance floor were transcribed by recording for each bout of dancing: the identity of the dancing bee, the duration of the dance bout, the number of waggle runs in the dance bout, and the number of followers per dance circuit. A dance bout was an instance of waggle dancing which consisted of several complete dance circuits with waggle runs, which ended when the focal bee ceased to dance for more than 60 sec. Only dance bouts performed by marked bees for the feeder-dish food source and consisting of at least three waggle runs were used for analysis. Any bouts during which the dancing bee moved out of the visible area in the video were not used for analysis. Bout duration was measured as the time between the start of the first waggle run to the end of the last waggle run. Dance followers were identified as those workers that remained near the dancer and oriented their heads toward the dancer as she moved through a dance circuit. The number of dance followers per circuit was determined by noting the number of dance followers during each circuit of the bout, summing these numbers for the entire bout, and then dividing by the number of circuits in the bout. This measure was used because the dance followers were not marked bees, and so while it was possible to keep track of individual followers within a single circuit, it was not possible to do so over the course of an entire bout of dancing. This measure thus reflects the relative amount of attention each bout received but cannot be used to estimate how many individual bees followed each dance. Video transcriptions were combined with records of behavior at the feeder to yield the following variables for analysis for each trial: 1) number of recruits captured at the feeder, 2) number of dance bouts, 3) number of waggle runs, 4) number of marked bees that engaged in waggle dancing, 5) mean number of dance bouts per dancer, 6) mean dance-bout duration, 7) mean waggle-run frequency (number of waggle runs divided by the bout duration, in minutes), 8) mean number of followers per dance circuit, 9) number of captured recruits per dance bout, and 10) number of captured recruits per waggle run. Trials during which there were less than five total dance bouts (due to poor weather conditions), were excluded from analysis. Colony 2010A was excluded from analyses of recruitment and number of dances (above variables 1, 2, 3, 4, 5, 9, and 10) because for this colony recruits were not captured at the feeder, creating positive feedback that would have affected the number of dances and thus recruitment. Colony 2010B was also excluded from analyses of recruitment (above variables 1, 9, and 10) because there were not enough recruits captured to make useful comparisons between treatments.

Transcription of these data allowed for analyses of effects of dance-compound treatment (versus solvent control) on measures of dance intensity (above variables 2 through 7) and on measures of dance efficacy (above variables 8 through 10), as well as establishing the effect of dance compounds on recruitment (above variable 1) in the absence of positive feedback from recruited bees. Because previous studies using similar substance-introduction techniques indicated peak recruitment effects 10 to 15 min following dance-compound introduction [44], the analyses here focus on the final fifteen minutes of each trial (Minute 30–45), i.e., between 15 and 30 min after substance introduction, unless otherwise noted. Statistical validation of the effect of the waggle-dance compounds on all dependent variables was obtained using linear mixed model procedures (Proc Mixed, SAS version 9.2) to account for lack of independence from repeated sampling of each colony. Linear mixed models used colony as a random factor and substance (solvent control or dance compounds) and time of day (morning, midday, afternoon) as fixed factors. Interaction effects between fixed factors are reported only where they were significant, otherwise they were eliminated from the model. Type III F-tests were used to test for fixed effects; degrees of freedom were estimated using the Satterthwaite method. Simultaneous tests of differences of least square means were conducted with p-values adjusted for multiple comparisons, which are reported where significant or where pair-wise comparisons are relevant. Model assumptions were checked by visual inspection of histograms of residuals, boxplots of the residuals, and scatterplots of residuals versus predicted values (following [48]). When necessary, response variables were transformed using the Box-Cox procedure (Minitab version 15), with the transformation function noted in the results.

## Results

### Number of recruits captured at the feeder

**Figure 1:**
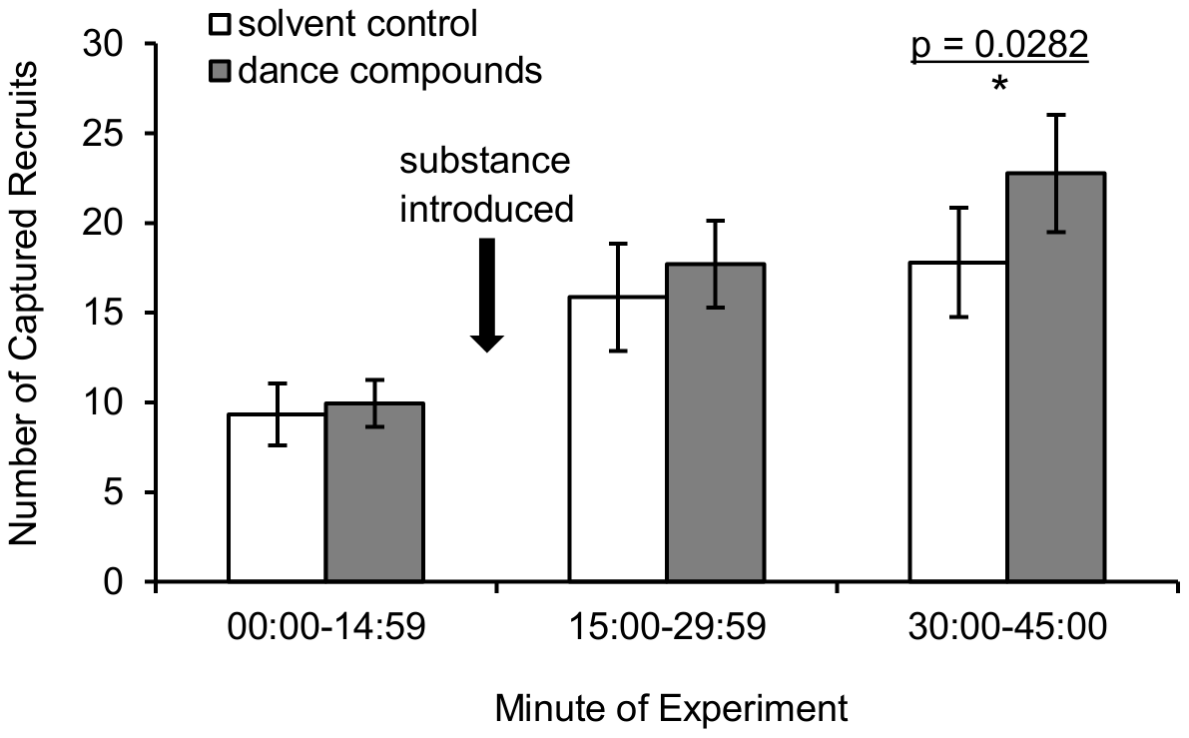
Number of recruits captured at the feeder following exposure to dance compounds versus solvent control. During 45-minute experiments either a mixture of the waggle-dance compounds or a pure-solvent control was introduced into the hive at Minute 15. Significantly more recruits were captured during Min 30–45 of dance-compound replicates than during solvent-control replicates (linear mixed model, Type III F = 5.40, df  = 1,26.0, p = 0.0282) when time of day and repeated sampling of colonies is taken into account. Heights of bars represent untransformed means of 15 solvent-control and 17 dance-compound trials; error bars represent the standard errors of these means.

The type of substance introduced (waggle-dance compounds or solvent control) significantly affected the number of recruits captured at the feeder 30 min after exposure (Figure 1; linear mixed model using recruits arrivals for Min 30–45 as dependent, colony as random effect, and substance and time of day as fixed effects; for substance: Type III F = 5.40, df  = 1,26.0, p = 0.0282; for time of day: Type III F = 19.40, df  = 2,26.0, p<0.0001; for substance*time of day interaction: Type III F = 0.70, d.f.  = 2,26.0, p = 0.5053). The number of captured recruits was transformed prior to statistical analysis using the Box-Cox procedure (lambda  = 0.450) to yield approximately normal distributions (Anderson-Darling normality tests, all p>0.579) with equal variance across treatments (Levene's Test, p = 0.567).

### Measures of colony waggle-dance intensity

**Figure 2:**
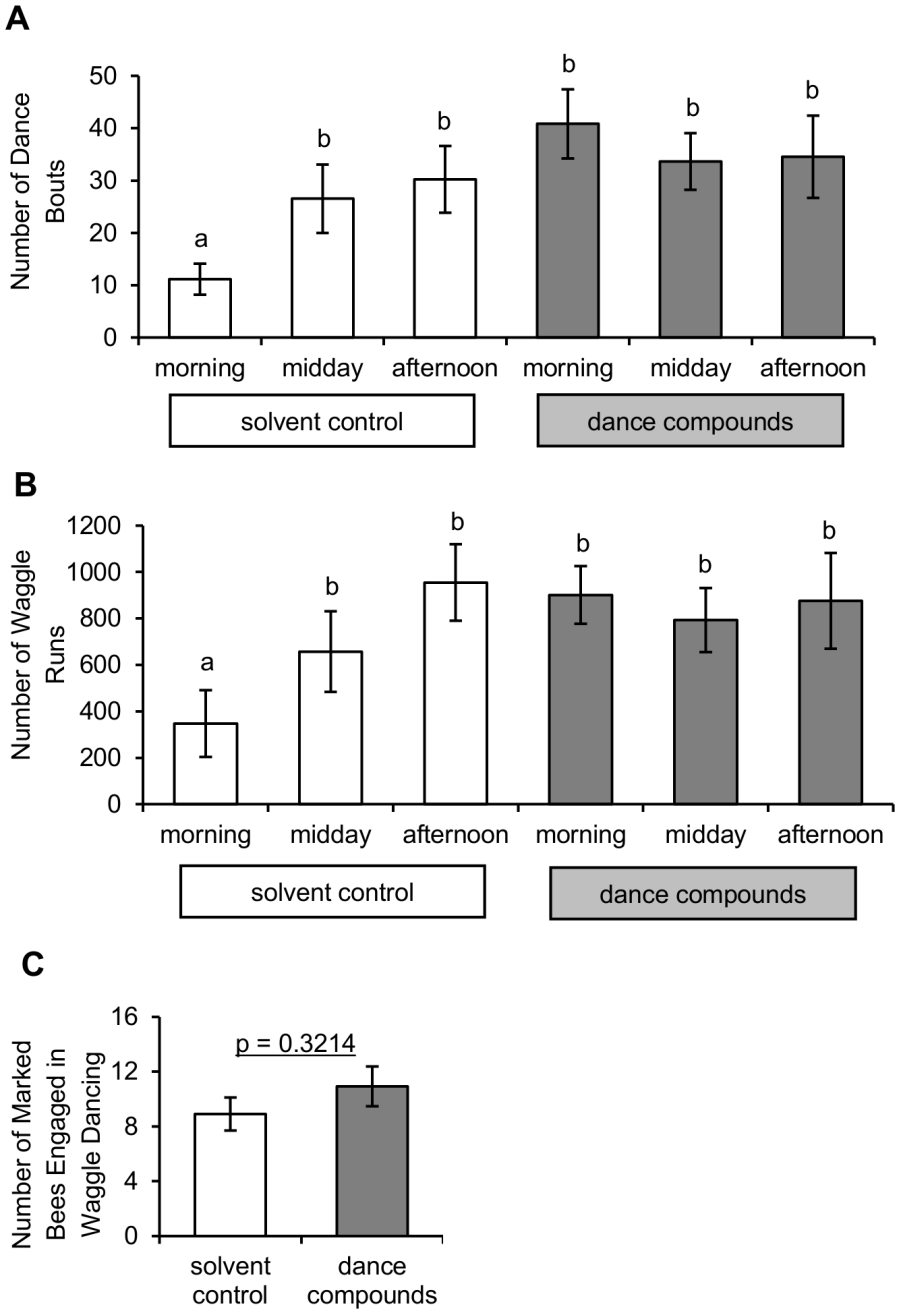
Colony waggle-dance intensity measures following exposure to waggle-dance compounds versus solvent control. The number of waggle-dance bouts (A) and number of waggle runs (B) was significantly higher in dance-compound versus solvent-control trials (linear mixed models; Type III F = 26.66, 6.60; p<0.0001, 0.0139; d.f.  = 1,39; respectively). There was a significant interaction effect between substance type and the time of day during which trials were conducted; fewer dance bouts and waggle runs were observed during morning control trials than during any other type of trial (letters represent significant differences of pairwise comparisons using Tukey-Kramer adjusted p-values, see text for statistics). (C) Dance-compound introduction did not have a significant effect on the number of marked bees engaging in waggle dance behavior (p-value represents a Type III F-test from a linear mixed model, see Table 1). Heights of bars represent untransformed means of 47 trials (A), 49 trials (B), and 44 trials (C); error bars represent the standard errors of these means.

Three measures of the intensity of waggle-dancing within colonies were examined for an effect of dance-compound introduction: number of waggle-dance bouts, number of waggle runs, and number of bees engaged in waggle dancing. The number of waggle-dance bouts was affected by dance-compound introduction (Figure 2 A; Table 1, linear mixed model using the number of dance bouts during Minute 30–45 as the response, colony as a random effect, and substance and time of day as fixed effects). Dance-compound introduction resulted in a highly significant increase in the number of dance bouts versus the solvent control (t = 5.16, d.f.  = 39, Tukey-Kramer adjusted p<0.0001). A significantly lower number of dance bouts was observed in morning replicates of the experiment versus the midday and afternoon (t = 4.03 and 4.44, d.f.  = 39, Tukey-Kramer adjusted p = 0.0007 and 0.0002). There was a significant interaction between substance introduced and time of day in that the number of dance bouts was significantly lower during morning replicates involving the solvent control than any other combination of time period and substance type (Figure 2 A; differences of least squares means between morning*ether and all five other combinations, all t>4.77, d.f.  = 39, all Tukey-Kramer adjusted p<0.0003; no other pairwise differences were significant after adjusting for multiple comparisons). The number of dance bouts was transformed using the Box-Cox procedure (lambda  = 0.449) to yield approximately normal distributions (Anderson-Darling normality tests, all p>0.254) with equal variance across treatments (Levene's Test, p = 0.374).

**Table 1:** Linear mixed model results for colony measures of waggle-dance intensity.

Response variable	Factor	Factor type	d.f.	Test statistic	P-value
Number of dance bouts	substance	fixed (a = 2)	1,39.0	F = 26.66	p<0.0001**
	time of day	fixed (a = 3)	2,39.0	F = 11.27	p = 0.0001**
	substance*time of day	(interaction)	2,39.0	F = 6.15	p = 0.0048**
	colony	random (a = 3)	–	Z = 0.99	p = 0.1604
Total number of waggle runs	substance	fixed (a = 2)	1,41.0	F = 6.60	p = 0.0139*
	time of day	fixed (a = 3)	2,41.0	F = 15.52	p<0.0001**
	substance*time of day	(interaction)	2,41.0	F = 4.52	p = 0.0168*
	colony	random (a = 3)	–	Z = 0.99	p = 0.1612
Number of bees engaged in waggle dancing	substance	fixed (a = 2)	1,38.1	F = 1.01	p = 0.3214
	time of day	fixed (a = 3)	1,38.3	F = 0.82	p = 0.4492
	colony	random (a = 3)	–	Z = 0.90	p = 0.1834

* indicates a significant p-value (p<0.05), ** indicates a highly significant p-value (p<0.01).

The number of waggle runs was significantly affected by the substance introduced (Figure 2 B; Table 1, linear mixed model using the number of waggle runs during Min 15–45 as the response, colony as a random effect, and substance and time of day as fixed effects). Dance-compound introduction resulted in a significant increase in the number of waggle runs versus the solvent control (t = 2.57, d.f.  = 41, Tukey-Kramer adjusted p = 0.0139). A significantly lower number of waggle runs was observed in morning replicates of the experiment versus the midday and afternoon (t = 3.77 and 5.54, d.f.  = 41, Tukey-Kramer adjusted p = 0.0015 and p<0.0001). There was a significant interaction between substance introduced and time of day in that the number of waggle runs was significantly lower during morning replicates involving the solvent control than any other combination of time period and substance type (differences of least squares means between morning/ether and all five other combinations, all t>3.65, d.f.  = 41, all Tukey-Kramer adjusted p<0.0091; no other pairwise differences were significant after adjusting for multiple comparisons). The number of waggle runs was transformed using the Box-Cox procedure (lambda  = 0.337) to yield approximately normal distributions (Anderson-Darling normality tests, all p>0.080) with equal variance across treatments (Levene's Test, p = 0.144).

The number of bees engaged in waggle dancing was not significantly affected by substance introduced, time of day, or colony (Figure 2 C; Table 2, linear mixed model using the number of individual bees that danced at least once during Min 15–45 as the response, colony as a random effect, and substance and time of day as fixed effects). The number of bees engaged in waggle dancing was transformed using the Box-Cox procedure (lambda  = −0.449) to yield approximately normal distributions for each treatment (Anderson-Darling normality tests, all p>0.174) with equal variances between the treatments (Levene's Test, p = 0.623).

**Table 2:** Linear mixed model results for individual measures of waggle-dance intensity.

Response variable	Factor	Factor type	d.f.	Test statistic	P-value
Mean number of dance bouts per dancer	substance	fixed (a = 2)	1,38.1	F = 4.10	p = 0.0499*
	time of day	fixed (a = 3)	2,38.2	F = 0.70	p = 0.5047
	colony	random (a = 3)	–	Z = 0.93	p = 0.1759
Mean dance-bout duration	substance	fixed (a = 2)	1,57.1	F = 0.36	p = 0.5483
	time of day	fixed (a = 3)	2,57.4	F = 4.80	p = 0.0118*
	colony	random (a = 4)	–	Z = 1.14	p = 0.1274
Mean waggle-run frequency	substance	fixed (a = 2)	1,57.1	F = 0.00	p = 0.9796
	time of day	fixed (a = 3)	2,57.5	F = 1.15	p = 0.3239
	colony	random (a = 4)	–	Z = 1.12	p = 0.1310

* indicates a significant p-value (p<0.05).

### Measures of individual waggle-dance intensity

**Figure 3:**
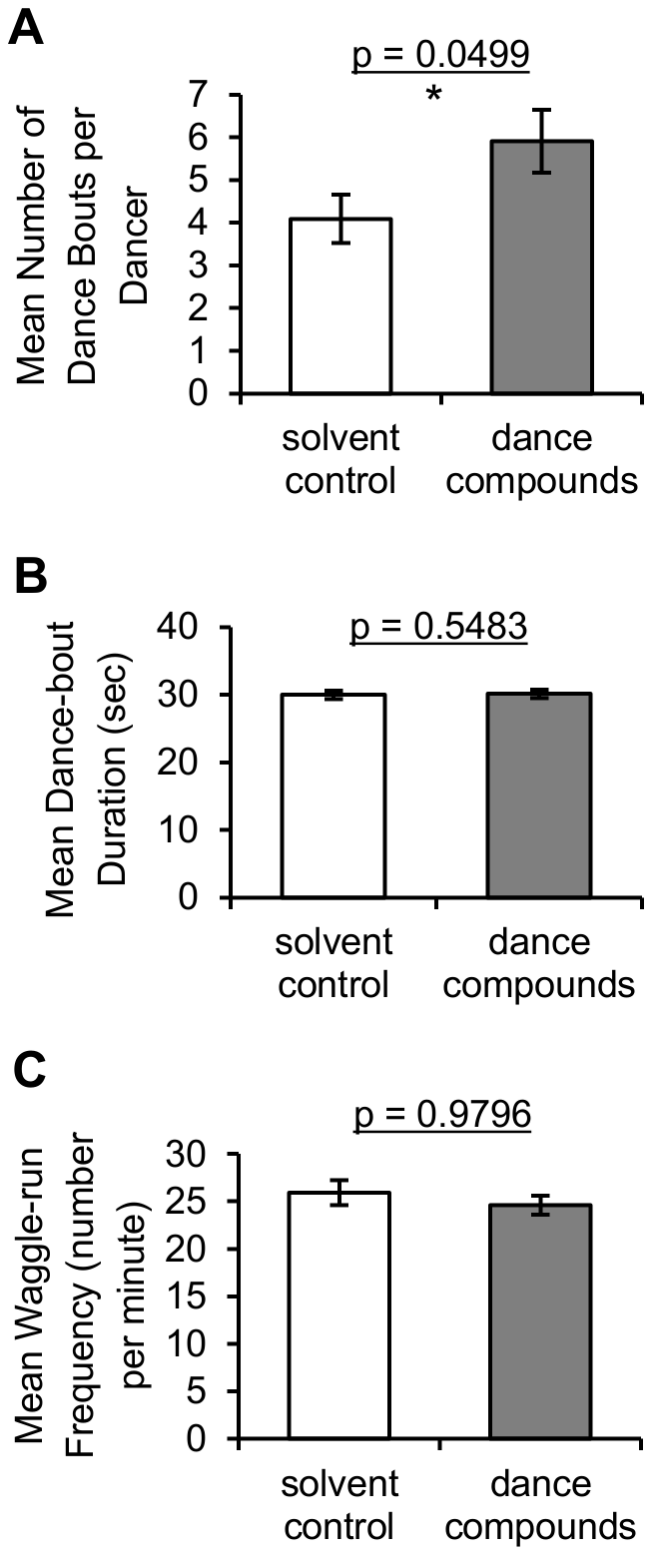
Individual waggle-dance intensity measures following exposure to waggle-dance compounds versus solvent control. (A) The mean number of waggle-dance bouts per dancer was marginally significantly higher in dance-compound versus solvent-control trials (linear mixed models; Type III F = 4.10; p = 0.0499; d.f.  = 1,38.1). Dance-compound introduction did not have a significant effect on (B) the mean dance-bout duration or (C) the mean waggle-run frequency (p-values represent Type III F-tests from linear mixed models, see Table 2). Heights of bars represent untransformed means of 44 trials (A), and 64 trials (B, C); error bars represent the standard errors of these means.

Three measures of waggle-dance intensity for individual dancing bees were examined for an effect of dance-compound introduction: mean number of dance bouts per dancer, mean dance-bout duration, and mean waggle-run frequency. The mean number of dance bouts per dancer, but not the other two measures, was affected by introduction of waggle-dance compounds versus the solvent control (Figure 3; Table 2, linear mixed models using each variable during Min 30–45 as the response, colony as random effect, and substance and time of day as fixed effects). A marginally significantly larger number of dance bouts per dancer was observed in dance-compound versus solvent-control trials (t = 2.03, d.f.  = 38.1, Tukey-Kramer adjusted p = 0.0499). None of these variables was significantly affected by colony or time of day, with the exception of bout duration, which was shorter in the morning versus midday and afternoon trials (t = 2.47, 3.03, d.f.  = 57.8, 57.6, Tukey-Kramer adjusted p = 0.0434, 0.0101). Both bout duration and waggle-run frequency were approximately normally distributed for each treatment (Anderson-Darling normality tests, all p>0.149) with equal variances between the treatments (Levene's Tests, all p>0.473). Dance bouts per dancer was transformed using the Box-Cox procedure (lambda  = 0) to yield approximately normal distributions for each treatment (Anderson-Darling normality tests, all p>0.296) with equal variances between the treatments (Levene's Test, p = 0.975).

### Measures of waggle-dance efficacy

Measures of waggle-dance efficacy examined for an effect of dance-compound introduction were the mean number of followers per waggle-dance circuit, the number of captured recruits per waggle run, and the number of captured recruits per dance bout. The mean number of followers per circuit was not significantly affected by introduction of waggle-dance compounds versus the solvent control, time of day, or colony (Figure 3 A; Table 2, linear mixed models using mean number of followers per dance circuit during Min 30–45 as the response, colony as random effect, and substance and time of day as fixed effects). The mean number of followers per dance circuit was approximately normally distributed for each treatment (Anderson-Darling normality tests, all p>0.165) with equal variance between treatments (Levene's Test, all p = 0.414).

**Table 3:** Linear mixed model results for measures of waggle-dance efficacy.

Response variable	Factor	Factor type	d.f.	Test statistic	P-value
Mean number of followers per dance circuit	substance	fixed (a = 2)	1,57.4	F = 0.08	p = 0.7835
	time of day	fixed (a = 3)	2,58.3	F = 0.72	p = 0.4895
	colony	random (a = 4)	–	Z = 0.87	p = 0.1922
Number of captured recruits per waggle run	substance	fixed (a = 2)	1,29.2	F = 0.04	p = 0.8479
	time of day	fixed (a = 3)	2,29.0	F = 4.09	p = 0.0272*
	colony	random (a = 2)	–	Z = 0.50	p = 0.3086
Number of captured recruits per dance bout	substance	fixed (a = 2)	1,29.3	F = 0.23	p = 0.6359
	time of day	fixed (a = 3)	2,29.1	F = 10.55	p = 0.0004**
	colony	random (a = 2)	–	Z = 0.39	p = 0.3486

* indicates a significant p-value (p<0.05), ** indicates a highly significant p-value (p<0.01).

Neither the number of captured recruits per dance bout nor the number of captured recruits per waggle run (n = 34 trials) was affected by dance-compound introduction (Figure 3 B, C; Table 2, linear mixed models using the number of captured recruits during Min 30–45 divided by the number of dances performed during Min 30–45, and number of arriving recruits during Min 15–45 divided by the number of waggle runs performed during Min 15–45 as the responses, colony as a random effect, and substance and time of day as fixed effects). These variables were also not significantly affected by colony, but were both affected by time of day. The number of recruits per dance was greater in the afternoon than at any other time of day (t = 3.33 and 4.37, d.f.  = 29, Tukey-Kramer adjusted p = 0.0065 and 0.0004). The number of recruits per waggle run was greater in the afternoon than midday (t = 2.67, d.f.  = 29, Tukey-Kramer adjusted p = 0.032), with a marginal but insignificant difference between afternoon and morning (t = 2.19, d.f.  = 29, Tukey-Kramer adjusted p = 0.0904). Both variables were transformed using the Box-Cox procedure (lambda  = −0.224 and −0.786, respectively) to yield normal distributions (Anderson-Darling normality tests, all p>0.263) with equal variance across treatments (Levene's Tests, all p>0.558).

## Discussion

**Figure 4:**
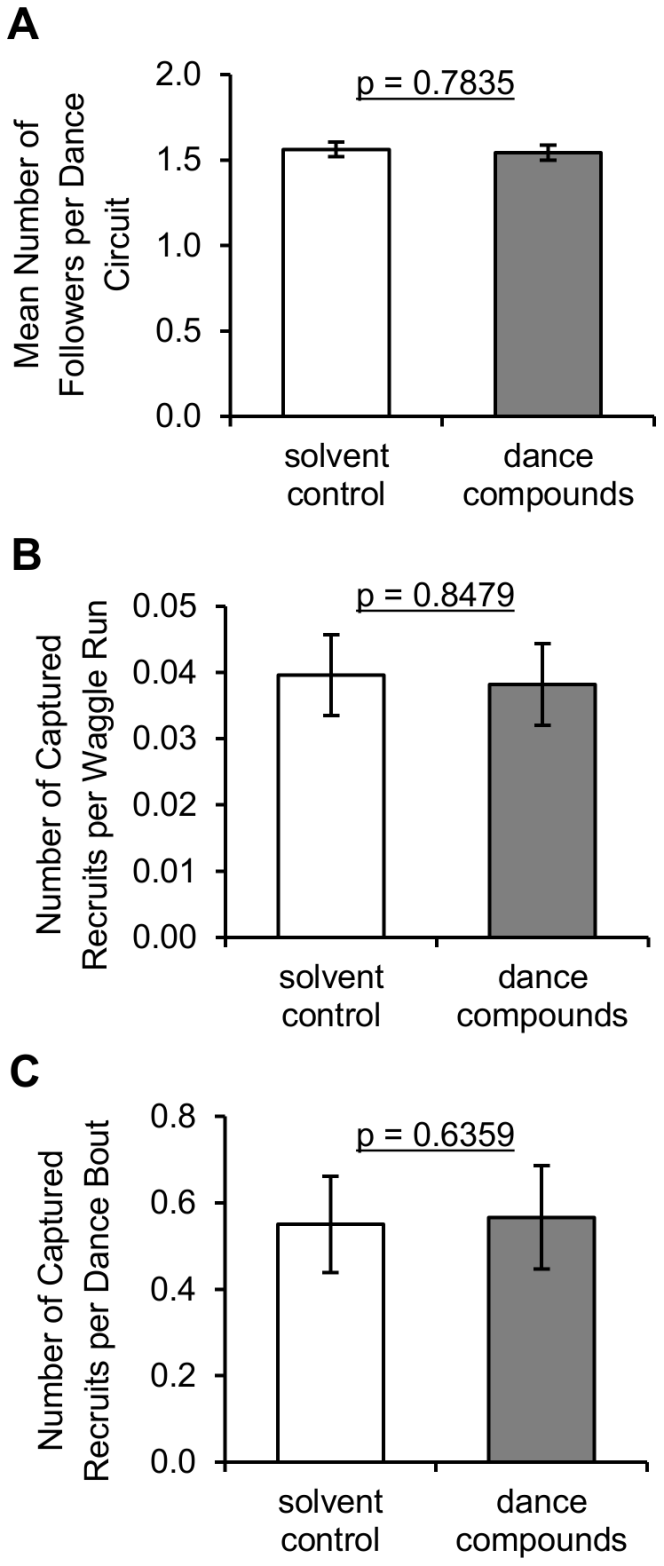
Dance efficacy measures following exposure to waggle-dance compounds versus solvent control. Dance-compound introduction had no effect on (A) the mean number of followers per dance circuit, (B) the number of captured recruits per waggle run, and (C) the number of captured recruits per dance bout (p-values represent Type III F-tests from linear mixed models, see Table 3). Heights of bars represent untransformed means of 64 trials (A) or 34 trials (B and C); error bars represent the standard errors of these means.

The results of this experiment show that introduction into the hive of the hydrocarbons emitted by waggle-dancing bees increases recruitment of foragers to a food source (versus the solvent control, Figure 1). The observed recruitment effect was not due to the compound-introduction procedure or to positive feedback among recruits because results are compared to a solvent-control introduction and recruits were captured before they could return to the hive. These results address the first goal of this study by showing that the dance hydrocarbons not only reactivate experienced foragers [44], but are also involved in recruitment to food sources via the waggle dance. This suggests that the waggle-dance hydrocarbons play an important role in the honey bee foraging-communication system. With respect to the second goal of this study, analysis of marked foragers' behavior on the hive dance floor shows that introduction of the dance hydrocarbons had little effect on the intensity of each waggle dance (Figure 3 B–C) but did increase colony signal intensity in that it increased the amount of waggle-dancing within the hive (Figure 2 A–B). This increase in colony signal intensity appears to have been caused by stimulating foragers to dance more often (Figure 3A) rather than stimulating additional foragers to participate in dance behavior (Figure 2 C). Analysis of foragers' behavior at the feeder and on the hive dance floor shows that hydrocarbon introduction had little effect on dance efficacy as measured by the number of dance followers (Figure 4 A) or recruit arrivals at the feeder per dance bout or waggle run (Figure 4 B–C). These results together provide support for the hypothesis that the behavioral mechanism by which the waggle-dance hydrocarbons stimulate recruitment is to increase the intensity of dance signaling and no support for the hypothesis that the dance hydrocarbons increase the recruitment effectiveness of each dance.

Before generalizing these conclusions to the natural functioning of the waggle-dance hydrocarbons, I must note two constraints of the experimental design. First, during these experiments what I assume to be a large amount of the dance compounds relative to natural levels was introduced into the hive, effectively flooding the dance floor with a “super-normal” signal. Thus, not only may the magnitude of the effect of these compound introductions on recruitment not be indicative of a natural level of response, but any natural responses to these compounds which relies on detection of the point source emitting these compounds (i.e., an individual waggle-dancing bee) would not have been detected. Second, there may be aspects of dancing and recruitment behavior that were not measured, but which might also be affected by exposure to the dance compounds. For example, the experimental design did not allow for individual tracking of dance followers or nectar receiver bees. These constraints make it incorrect to rule out from the results presented here the hypothesis that the waggle-dance compounds increase the recruitment effectiveness of each dance. The results of the experiments presented here should instead be interpreted as support for the hypothesis that the waggle-dance compounds increase recruitment of foragers to food sources by stimulating waggle-dancing.

The results presented here suggest that the waggle–dance hydrocarbons are an important part of the complex interplay of signals and cues which produce adaptive colony foraging behavior. The waggle-dance hydrocarbons appear to stimulate two key processes that allow honey bee colonies to successfully exploit floral food sources, recruitment through the waggle dance (this study) and reactivation of unemployed experienced foragers [44]. In nature, these two processes would work in tandem to create through positive feedback a rapid increase in recruitment to a profitable food source. In the present study, positive feedback was constrained by the capture of recruits at the food source but a significant effect of compound introduction on recruitment was still observed (Figure 1). Capture of recruits likely explains why the effect of waggle-dance compound introduction on bee arrivals at a food source was less pronounced here than in a previous study, where positive feedback following compound introduction led to approximately 100% increase in recruitment over the same 30 min period [44]. The stimulation of recruitment observed here was apparently driven by the unusually high morning dance activity of experienced bees when exposed to dance compounds (Figure 2 A–B) because measures of per-dance intensity (Figure 3 B–C) and per-dance efficacy (Figure 4) did not show strong effects of substance. The key receivers of these semiochemicals appear from these results to be both active and inactive foragers rather than potential recruits. The precise nature of the receivers' behavioral response (e.g., decrease in their threshold for dancing or increase in their rate of foraging trips) remains unclear from the data presented. That introduction of dance compounds increased normally low morning levels of dancing to levels indistinguishable from normally high afternoon levels (Figure 2 A–B) suggests that these semiochemicals function to stimulate recruitment following periods of inactivity (e.g. night hours or poor weather). That introduction of dance compounds did not change dance levels in the midday or afternoon trials (Figure 2) suggests that these semiochemicals have a less significant role once foraging is at its peak. Whether the waggle-dance hydrocarbons should be considered a signal or cue remains ambiguous pending identification of their physiological source.

Transmission of the hydrocarbons from waggle dancers to receivers could occur by bee-to-bee contact and/or by diffusion through the air. Both of these modes of transmission were possible during this experiment, as bees had free access to the glass slide upon which the substances were introduced. Many low-volatility honey bee semiochemicals, such as queen pheromones and nestmate recognition cues [49], are transmitted or detected by contact between the body parts of nestmates. Workers in this experiment did not interact in any obvious way with the slide (anecdotal observations), as might be expected if contact is the only mode of transmission. However, this might have been due to lack of appropriate context (i.e., no bee body was present) rather than an indication that contact is not an important means of transmission. Returning foragers could have picked up from the treatment slide onto their tarsi heightened levels of dance hydrocarbons, which were then detected by other experienced foragers with which they came into contact. However, contact between marked foragers was rarely observed; less than 1% of the 4863 bouts of waggle dancing included one or more marked bees as followers. In the absence of an obvious contact pathway from the introduced compounds to the active foragers, whose behavior was here effected by these compounds, it seem reasonable to conclude that diffusion through the air plays at least some role in the transmission of the hydrocarbons from waggle dancers to the receivers of these semiochemicals. Airborne transmission further seems likely given that the dance hydrocarbons have been detected in the air surrounding active foragers in both the hive [43] and field [47], and blowing heated air containing the hydrocarbons has been shown to affect bees' behavior [43].

## References

[1] Dornhaus A, Chittka L (2001) Food alert in bumblebees (*Bombus terrestris*): possible mechanisms and evolutionary implications. Behavioral Ecology and Sociobiology 50(6): 570–576.

[2] Dornhaus A, Cameron S (2003) A scientific note on food alert in *Bombus transversalis* . Apidologie 34(1): 87–88.

[3] Dornhaus A, Chittka L (2004) Why do honey bees dance? Behavioral Ecology and Sociobiology 55(4): 395–401.

[4] Lindauer M, Kerr WE (1960) Communication between the workers of stingless bees. Bee World 41: 29–41.

[5] Kerr WE (1994) Communication among Melipona workers (Hymenoptera: Apidae). Journal of insect behaviour 7(1): 123–128.

[6] Nieh JC, Contrera FA, Yoon RR, Barreto LS, Imperatriz-Fonseca VL (2004) Polarized short odor-trail recruitment communication by a stingless bee, *Trigona spinipes* . Behavioral Ecology and Sociobiology 56(5): 435–448.

[7] Free JB, Williams IH (1972) The role of the Nasanov gland pheromone in crop communication by honeybees (*Apis mellifera L.*). Behaviour 41: 314–318.

[8] Ferguson AW, Free JB (1979) Production of forage-marking pheromone by the honeybee. Journal of Apicultural Research 18: 128–135.

[9] Cameron SA (1981) Chemical signals in bumblebee foraging. Behavioral Ecology and Sociobiology 9: 257–260.

[10] Free JB, Williams I, Pickett JA, Ferguson AW, Martin AP (1982) Attractiveness of (Z)-11-eicosen-1-ol to foraging honeybees. Journal of Apicultural Research 21: 151–156.

[11] Free JB, Williams IH (1983) Scent-marking of flowers by honeybees. Journal of Apicultural Research 22: 86–90.

[12] Schmitt U, Bertsch A (1990) Do foraging bumblebees scentmark food sources and does it matter? Oecologia 82: 137–144. 28313149 10.1007/BF00318545

[13] Schmitt U, Lubke G, Francke W (1991) Tarsal secretion marks food sources in bumblebees (Hymenoptera: Apidae). Chemoecology 2: 35–40.

[14] Vallet A, Cassier P, Lensky Y (1991) Ontogeny of the fine structure of the mandibular glands of the honeybee (*Apis mellifera L.*) workers and the pheromonal activity of 2-heptanone. Journal of Insect Physiology 37: 789–804.

[15] Giurfa M (1993) The repellent scent-mark of the honeybee *Apis mellifera ligustica* and its role as communication cue during foraging. Insectes Sociaux 40: 59–67.

[16] Oldham NJ, Billen J, Morgan ED (1994) On the similarity of the Dufour gland secretion and the cuticular hydrocarbons of some bumblebees. Physiological entomology 19(2): 115–123.

[17] Stout JC, Goulson D, Allen JA (1998) Repellent scentmarking of flowers by a guild of foraging bumblebees (Bombus spp.). Behavioral Ecology and Sociobiology 43: 317–326.

[18] Goulson D, Stout JC, Langley J, Hughes WOH (2000) The identity and function of scent marks deposited by foraging bumblebees. Journal of Chemical Ecology 26: 2897–2911.

[19] Goulson D, Chapman JW, Hughes WO (2001) Discrimination of unrewarding flowers by bees; direct detection of rewards and use of repellent scent marks. Journal of Insect Behavior 14(5): 669–678.

[20] Stout JC, Goulson D (2001) The use of conspecific and interspecific scent marks by foraging bumblebees and honeybees. Animal behaviour 62(1): 183–189.

[21] Jarau S, Hrncir M, Ayasse M, Schulz C, Francke W, et al (2004) A stingless bee (*Melipona seminigra*) marks food sources with a pheromone from its claw retractor tendons. Journal of chemical ecology 30(4): 793–804. 15260224 10.1023/b:joec.0000028432.29759.ed

[22] Hrncir M, Jarau S, Zucchi R, Barth FG (2004) On the origin and properties of scent marks deposited at the food source by a stingless bee, *Melipona seminigra* . Apidologie 35(1): 3–14.

[23] Saleh N, Scott AG, Bryning GP, Chittka L (2007) Distinguishing signals and cues: bumblebees use general footprints to generate adaptive behaviour at flowers and nest. Arthropod-Plant Interactions 1(2): 119–127.

[24] Renner MA, Nieh JC (2008) Bumble bee olfactory information flow and contact-based foraging activation. Insectes sociaux 55(4): 417–424.

[25] Von Frisch K (1967) The dance language and orientation of bees. Cambridge: Harvard University Press.

[26] Dornhaus A, Chittka L (1999) Evolutionary origins of bee dances. Nature 401: 38.

[27] Seeley TD (1998) Thoughts on information and integration in honey bee colonies. Apidologie 29(1–2): 67–80.

[28] Biesmeijer JC, Slaa EJ (2004) Information flow and organization of stingless bee foraging. Apidologie 35(2): 143–157.

[29] Wenner AM, Johnson DL (1967) Honeybees: do they use direction and distance information provided by their dancers? Science 158(3804): 1072. 6058349 10.1126/science.158.3804.1072

[30] Wenner AM, Wells PH, Johnson DL (1969) Honey bee recruitment to food sources: olfaction or language? Science 164(3875): 84–86. 5773718 10.1126/science.164.3875.84

[31] Gould JL, Henerey M, MacLeod MC (1970) Communication of direction by the honey bee: review of previous work leads to experiments limiting olfactory cues to test the dance language hypothesis. Science 169(3945): 544–554. 5426774 10.1126/science.169.3945.544

[32] Lindauer M (1971) The functional significance of the honeybee waggle dance. American Naturalist 105: 89–96.

[33] Gould JL (1976) The dance-language controversy. Quarterly Review of Biology 51: 211–244. 785523 10.1086/409309

[34] Kirchner WH, Grasser A (1998) The significance of odor cues and dance language information for the food search behavior of honeybees (Hymenoptera: Apidae). Journal of insect behavior 11(2): 169–178.

[35] Fernández PC, Farina WM (2001) Changes in food source profitability affect Nasonov gland exposure in honeybee foragers *Apis mellifera L.* Insectes sociaux. 48(4): 366–371.

[36] Farina WM, Grüter C, Díaz PC (2005) Social learning of floral odours inside the honeybee hive. Proceedings of the Royal Society B: Biological Sciences 272(1575): 1923–1928. 16191598 10.1098/rspb.2005.3172 PMC1559887

[37] Farina WM, Grüter C, Acosta L, Mc Cabe S (2007) Honeybees learn floral odors while receiving nectar from foragers within the hive. Naturwissenschaften 94(1): 55–60. 17021915 10.1007/s00114-006-0157-3

[38] Díaz PC, Grüter C, Farina WM (2007) Floral scents affect the distribution of hive bees around dancers. Behavioral Ecology and Sociobiology 61(10): 1589–1597.

[39] Arenas A, Fernández VM, Farina WM (2007) Floral odor learning within the hive affects honeybees' foraging decisions. Naturwissenschaften 94(3): 218–222. 17119909 10.1007/s00114-006-0176-0

[40] Reinhard J, Srinivasan MV, Zhang S (2004) Olfaction: scent-triggered navigation in honeybees. Nature 427(6973): 411. 10.1038/427411a 14749818

[41] Beekman M (2005) How long will honey bees (*Apis mellifera L.*) be stimulated by scent to revisit past-profitable forage sites? Journal of Comparative Physiology A 191(12): 1115–1120. 10.1007/s00359-005-0033-1 16049699

[42] Ribbands CR (1954) Communication between honeybees. I: the response of crop-attached bees to the scent of their crop. Proceedings of the Royal Entomological Society of London A 29(10–12): 141–144.

[43] Thom C, Gilley DC, Hooper J, Esch HE (2007) The scent of the waggle dance. PLoS Biology 5(9): e228 doi:10.1371/journal.pbio.0050228 17713987 10.1371/journal.pbio.0050228 PMC1994260

[44] Gilley DC, Kuzora JM, Thom C (2012) Hydrocarbons emitted by waggle-dancing honey bees stimulate colony foraging activity by causing experienced foragers to exploit known food sources. Apidologie 43(1): 85–94.

[45] Howard RW, Blomquist GJ (1982) Chemical ecology and biochemistry of insect hydrocarbons. Annual review of entomology 27(1): 149–172. 10.1146/annurev-ento-031620-071754 33417824

[46] Breed MD (1998) Recognition pheromones of the honey bee. Bioscience 48(6): 463–470.

[47] Schmitt T, Herzner G, Weckerle B, Schreier P, Strohm E (2007) Volatiles of foraging honeybees *Apis mellifera* (Hymenoptera: Apidae) and their potential role as semiochemicals. Apidologie 38(2): 164–170.

[48] West BT, Welch KB, Galecki AT (2006) Linear mixed models: a practical guide using statistical software. Boca Raton, Florida: Chapman & Hall/CRC Press. 348 p.

[49] Free JB (1987) Pheromones of social bees. Ithaca, New York: Cornell University Press. 192 p.

